# EP23 Symptomatic sarcoid myopathy: a rare extra-pulmonary manifestation of sarcoidosis

**DOI:** 10.1093/rap/rkaa052.022

**Published:** 2020-11-03

**Authors:** Saadia Sasha Ali, Mark Russell, James Galloway, Ioana Onac

**Affiliations:** King's College Hospital, London, United Kingdom

## Abstract

**Case report - Introduction:**

Sarcoidosis is a multisystem disorder of unknown aetiology that is characterised pathologically by the presence of non-caseating granulomata. The disease is known for its multitude of presentations and can affect almost any organ system. Symptomatic skeletal muscle involvement in sarcoidosis is infrequent and occurs in < 3% of all sarcoidosis patients. We present the case of a 47-year-old male with multisystem sarcoidosis involving his lungs, eyes, and liver, who presented to our tertiary sarcoid centre with proximal muscle weakness. This case is significant as it highlights the diagnostic challenges that can arise when muscle weakness occurs on a background of sarcoidosis.

**Case report - Case description:**

A 47-year-old gentleman presented to Rheumatology with a ten-year history of progressive lower extremity muscle weakness. He was known to have multisystem sarcoidosis affecting his lungs (diffuse interstitial lung disease), eyes (anterior uveitis) and liver (liver fibrosis). His sarcoidosis was initially diagnosed ten years beforehand, from confirmatory histology obtained via Endobronchial Ultrasound sampling.

He was previously a keen runner; however, he had observed a gradual decline in his ability to run. Over a period of two years his mobility further deteriorated, and he required two sticks to walk.

Physical examination revealed a waddling gait with wasting to his quadriceps bilaterally. He had reduced power of 2/5 on hip flexion on the Medical Research Council (MRC) muscle grading scale. There was no bulbar involvement and facial and upper extremity strength was normal.

His past medical history was also remarkable for anxiety and depression. There was no family history of muscle disease.

Serology revealed a Creatine Kinase (CK) of 773 IU/L (32-294 IU/L). He had an equivocal signal recognition particle (SRP) antibody result, which was later repeated and found to be negative. His EMG showed myopathic changes in his distal and proximal lower limb muscles with profuse spontaneous activity, indicating an active myopathic process.

MRI of his lower limbs showed symmetrical fatty infiltration in the distal semimembranosus and short head of biceps femoris muscles with no clear oedema. A muscle biopsy showed diffuse MHC Class 1 upregulation with nemaline rods.

Treatment with pulsed IV methylprednisolone was started, in addition to Mycophenolate Mofetil (MMF) as steroid therapy was not sufficient to suppress his disease. He had a reduction in his CK to 205 IU/L and no activity in his skeletal muscle on FDG-PET CT. His power improved to 3/5 on MRC grading.

**Case report - Discussion:**

Three distinct patterns of muscle involvement in sarcoidosis are recognised: chronic myopathy, nodular myopathy, and acute myopathy. Symptomatic muscle disease in sarcoidosis is rare and it is important to consider other potential aetiologies of a progressive myopathy, even in a patient with established multisystem sarcoidosis. This case is interesting as there was diagnostic difficulty in ascertaining the diagnosis, which potentially included a corticosteroid-induced myopathy, SRP necrotising myopathy, or even a nemaline myopathy.

Corticosteroid myopathy has a similar distribution to a sarcoid myopathy. However, the patient’s clinical phenotype, elevated muscle enzymes, EMG findings, and histological data favoured an inflammatory myositis.

SRP necrotising myopathy is characterised by rapidly progressive proximal muscle weakness with necrotic muscle fibres, scant inflammation, and a significant elevation in muscle enzymes, which were not seen in this patient.

The patient’s weakness was more insidious in onset, with diffuse inflammation on muscle biopsy. Nemaline rods were seen on biopsy, however these were only present in one area, which is atypical of a nemaline myopathy. Furthermore, the presence of many loculated fibres on biopsy and upregulation of MHC class 1 was more in keeping with a diagnosis of an inflammatory myopathy secondary to sarcoidosis, even in the absence of non-caseating granulomas on muscle biopsy.

There are no randomised controlled trials of treatments in sarcoid myopathy. While methotrexate is most used in steroid-recalcitrant myositis, the patient’s liver fibrosis preluded this therapy, thus MMF was trialled instead.

Co-existing inflammatory muscle disease with sarcoidosis has been documented infrequently in the literature. They both have overlapping symptoms with contrasting treatment strategies. In this patient, the muscle biopsy pointed to an idiopathic inflammatory myopathy (IIM) without granulomatous infiltration, it is intriguing to consider whether treatment of an IIM with intravenous immunoglobulin or rituximab would have resulted in better clinical outcomes.

**Case report - Key learning points:**

Key points:

1. Even though symptomatic muscle involvement in sarcoidosis is uncommon, a sarcoid myopathy should be suspected in symptomatic patients with known or suspected pulmonary or extrapulmonary sarcoidosis.
2. In patients without known sarcoidosis but with unexplained muscle symptoms, particularly in the setting of a multisystem illness, sarcoid myopathy should be considered in the differential diagnosis.
3. MRI and muscle biopsy are useful in distinguishing a sarcoid myopathy from a corticosteroid-induced myopathy as illustrated in this case.
4. Fluorine 18 fluorodeoxyglucose (FDG) PET/CT is sensitive for assessment of the inflammatory activity of sarcoidosis in any organ. In this patient, FDG-PET was useful in evaluating active sarcoid lesions and evaluating the therapeutic effects of Mycophenolate Mofetil on his sarcoid myopathy.
5. Although there is limited data to guide treatment in a sarcoid myopathy, Mycophenolate Mofetil can be used as an alternative to Methotrexate.

